# Inter-Metastatic Heterogeneity of Tumor Marker Expression and Microenvironment Architecture in a Preclinical Cancer Model

**DOI:** 10.3390/ijms22126336

**Published:** 2021-06-13

**Authors:** Jessica Kalra, Jennifer Baker, Justin Song, Alastair Kyle, Andrew Minchinton, Marcel Bally

**Affiliations:** 1 Experimental Therapeutics, BC Cancer Agency, Vancouver, BC V5Z 1L3, Canada; mbally@bccrc.ca; 2 Applied Research Centre, Langara, Vancouver, BC V5Y 2Z6, Canada; 3 Department Anesthesia Pharmacology and Therapeutics, University of British Columbia, Vancouver, BC V6T 1Z4, Canada; 4 Faculty of Pharmaceutical Sciences, University of British Columbia, Vancouver, BC V6T 1Z4, Canada; aim@bccrc.ca; 5 Integrative Oncology, BC Cancer Agency, Vancouver, BC V5Z 1L3, Canada; jbaker@bccrc.ca (J.B.); akyle@bccrc.ca (A.K.); 6 Chemical and Biomolecular Engineering Department, Vanderbilt University, Nashville, TN 37235, USA; justin.m.song@vanderbilt.edu; 7 Pathology and Laboratory Medicine, University of British Columbia, Vancouver, BC V6T 1Z4, Canada; 8 Nanomedicine Innovation Network, University of British Columbia, Vancouver, BC V6T 1Z4, Canada

**Keywords:** inter-metastatic heterogeneity, preclinical studies, multiplex immunohistochemistry, drug development, translational medicine, metastasis, chemoresistance

## Abstract

Background: Preclinical drug development studies rarely consider the impact of a candidate drug on established metastatic disease. This may explain why agents that are successful in subcutaneous and even orthotopic preclinical models often fail to demonstrate efficacy in clinical trials. It is reasonable to anticipate that sites of metastasis will be phenotypically unique, as each tumor will have evolved heterogeneously with respect to gene expression as well as the associated phenotypic outcome of that expression. The objective for the studies described here was to gain an understanding of the tumor heterogeneity that exists in established metastatic disease and use this information to define a preclinical model that is more predictive of treatment outcome when testing novel drug candidates clinically. Methods: Female NCr nude mice were inoculated with fluorescent (mKate), Her2/neu-positive human breast cancer cells (JIMT-mKate), either in the mammary fat pad (orthotopic; OT) to replicate a primary tumor, or directly into the left ventricle (intracardiac; IC), where cells eventually localize in multiple sites to create a model of established metastasis. Tumor development was monitored by in vivo fluorescence imaging (IVFI). Subsequently, animals were sacrificed, and tumor tissues were isolated and imaged ex vivo. Tumors within organ tissues were further analyzed via multiplex immunohistochemistry (mIHC) for Her2/neu expression, blood vessels (CD31), as well as a nuclear marker (Hoechst) and fluorescence (mKate) expressed by the tumor cells. Results: Following IC injection, JIMT-1mKate cells consistently formed tumors in the lung, liver, brain, kidney, ovaries, and adrenal glands. Disseminated tumors were highly variable when assessing vessel density (CD31) and tumor marker expression (mkate, Her2/neu). Interestingly, tumors which developed within an organ did not adopt a vessel microarchitecture that mimicked the organ where growth occurred, nor did the vessel microarchitecture appear comparable to the primary tumor. Rather, metastatic lesions showed considerable variability, suggesting that each secondary tumor is a distinct disease entity from a microenvironmental perspective. Conclusions: The data indicate that more phenotypic heterogeneity in the tumor microenvironment exists in models of metastatic disease than has been previously appreciated, and this heterogeneity may better reflect the metastatic cancer in patients typically enrolled in early-stage Phase I/II clinical trials. Similar to the suggestion of others in the past, the use of models of established metastasis preclinically should be required as part of the anticancer drug candidate development process, and this may be particularly important for targeted therapeutics and/or nanotherapeutics.

## 1. Introduction

Animal models are critical to the anticancer drug candidate development process; however, it has been established that preclinical animal studies are poor predictors of drug activity in the clinic [1,2,3,4,5,6,7,8,9]. It is widely agreed that subcutaneous tumor models are unable to recapitulate the complexity of cancer with respect to tumor heterogeneity, metastasis, or the influences of the local microenvironment, all of which are affected temporally following treatment. Orthotopic models of a variety of cancers such as breast, pancreatic, and lung cancers have been developed to better replicate tumor microenvironments. Some studies have even demonstrated that orthotopic tumors can copy the tumor architecture, cell morphology, and molecular characteristics of the comparable human tumor [10,11,12,13]. However, orthotopic models are also problematic in translational medicine. Treatments shown to be effective against orthotopic tumors are not necessarily effective against later stages of disease where the cancer has disseminated to multiple organs and tissues. Patient-derived xenografts (PDX) have become popular as a more predictive option however, not all patient tissues can be successfully established as PDX models [14]. These models are also labor-intensive, costly, and may have a significant lag between engraftment and first passage [15,16,17,18]. Furthermore, lag time contributes to slow growth, as well as rare and inconsistent metastasis [19] and PDX models change genetically and phenotypically with each passage, making them inherently poorly reproducible. There has been a long-recognized need to define experimental models of metastatic cancer that are robust, reproducible, and more predictive of clinical effects in patients typically enrolled in Phase I clinical trials [20,21]. Already, a significant amount of work has been carried out towards reaching these goals [20,21,22,23,24,25,26,27,28,29,30,31]. Collectively, these studies strongly suggest that variability in the effectiveness of drug candidates in a metastatic setting could be a result of heterogeneity in target expression or heterogeneity of the tumor microenvironment that may influence drug candidate distribution, drug activity and/or tissue resistance. 

Genomic studies have already shown that tumors are evolutionarily active, and metastatic lesions are genetically distinct entities [13,32,33,34,35]. This genetic heterogeneity is often ascribed as the underlying mechanism through which tumors develop therapeutic resistance [36,37,38,39]. However, the extent and the implications of genetic diversity need to be fully explored phenotypically as much of the data generated through genome-wide sequencing has yet to be confirmed at the protein level. From the perspective of drug candidate development, the effect of phenotypic heterogeneity on drug efficacy will be significant, as it will relate to the variable expression of the drug target or activity of targeted pathways.

Inter-metastatic heterogeneity of tumor cell markers is a critical consideration but equally important is the heterogeneity and complexity of the local microenvironments in which these tumors are found [40]. If we examine one highly heterogeneous characteristic of the microenvironment alone, the development and functionality of tumor vasculature, there are at least three considerations in the context of drug efficacy: (i) Within solid primary tumors, the erratic nature of tumor vascular growth creates a highly variable microenvironment that is characterized by gradients in oxygen and nutrient supply, waste disposal, and drug access. These gradients then influence characteristics such as gene expression (drug targets) and cell cycle status (drug sensitivity), which can ultimately impact the ability of cells to cope with microenvironmental and therapeutic stresses. (ii) Tumors attract new blood vessel growth from neighboring tissues [38,41]. This means that in a metastatic setting, the organ/tissue that permits cancer cell seeding and growth likely plays a significant role in the vascular microenvironment, and consequently could impact drug delivery, distribution, and efficacy [42,43,44,45,46,47,48,49,50]. (iii) Treatments can affect vessel growth, structure, and function, adding to the complexity of vascular heterogeneity, which will change as a function of time after treatment is initiated [51,52]. Regardless of the root cause of vascular heterogeneity (i.e., location of disease burden or therapeutic implications), when evaluating a drug candidate’s ability to reach and penetrate within a given tumor, at the very least, the vessel microenvironment must be considered, and it is insufficient to investigate in the context of just one site of tumor growth if the goal is to obtain meaningful therapeutic results in late-stage patients.

Considering that patients enrolled in clinical trials for the evaluation of candidate drugs have already been heavily pretreated and likely have late-stage metastatic disease, preclinical studies should attempt to model the phenotypic and microenvironmental tumor heterogeneity that would be seen in these patients. Novel cytotoxic and targeted anti-cancer drug candidates may show more promise in the clinic if the preclinical studies used to test their efficacy accounted for tumor and microenvironmental heterogeneity. Further, formulation strategies that rely on delivery systems such as lipid-based or polymer-based nanoparticles, could be designed and optimized to ensure activity regardless of regional variability. 

A better understanding of the intra-tumor, inter-tumor, and inter-metastatic heterogeneity of tumor marker expression, blood vessel density, and ECM components and their impacts on drugs would help to define the models that should be considered in these types of preclinical evaluations. In an attempt to characterize one such model and establish proof of principle, the observational studies described here compare the tumor architecture and microenvironment in lesions found in different organs. A multitude of microenvironmental and tumor markers were examined in a human breast cancer cell line, JIMT-1, established either as an orthotopic xenograft or as multiple systemic lesions that form after intracardiac injection of the cells. JIMT-1 is a Her2/neu-positive cell line with the ability to consistently form tumors in the lung, liver, brain, kidney, ovaries, and adrenal glands. The results demonstrate that the metastatic lesions which arise after the inoculation of this genetically stable cell line show inter-metastatic heterogeneity at the tissue level. While heterogeneity was seen in each of the markers surveyed, we report specifically on the heterogeneity of a widely studied tumor marker (Her2/neu) and a well-established blood vessel maker (CD31). This is the first systematic survey to semi-quantitatively describe the extent of heterogeneity of both of these markers in metastatic sites collected from multiple organs. This collection of observational data supports the conclusion that models of established metastasis such as the one employed, could be ideal for assessing the effects of a preclinical candidate drug. However, and more importantly, these studies add to the body of literature that continues to highlight the need to identify multiple cancer models that recapitulate complex elements of the microenvironment and more closely resemble important characteristics of aggressive disease seen in late-stage patients [2,4,19,39].

## 2. Results

### 2.1. Development of an Orthotopic and Established Metastasis Model Using mKate-Positive JIMT-1 Breast Cancer Cells

**Figure 1:**
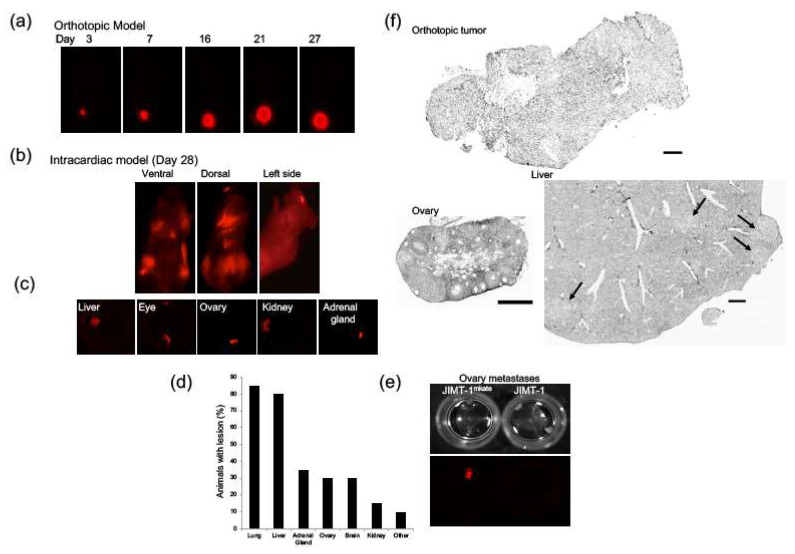
JIMT-1mKate cells (red) were inoculated in the mammary fat pad or left ventricle of female NCr nude mice. Subsequently, solid tumor (**a**) and metastatic (**b**) progression was followed using Maestro imaging. Organs excised from animals were imaged ex vivo using Maestro imaging (**c**). Disease consistently developed in the liver, lungs, kidney, ovaries, adrenal gland, and brain (**d**). Tumor-positive ovaries from animals inoculated with JIMT-1mKate and JIMT-1 cells were used as a positive and negative control respectively, to optimize Maestro imaging (**e**). Tumors and metastatic lesions were excised and subject to hematoxylin staining (**f**). Selected images show an orthotopic tumor (top bar = 250 µm), a tumor-infiltrated ovary (bottom, left, bar = 500 µm), and tumor nodules in the liver (arrows, bar = 250 µm).

JIMT-1mKate cells were inoculated into the mammary fat pad or left ventricle of female NCr animals to develop an orthotopic model and a model of established metastasis, respectively (*n* = 6). Animals were imaged using the Maestro in vivo imaging system over the course of 28 days. Mammary fat pad tumors reached an average of 500 mm^3^ at this time (Figure 1a). Figure 1b shows representative maestro images of the dorsal, ventral, and lateral views of a selected animal 19 days post-intracardiac inoculation of the JIMT-1mKate cells. Imaging illustrated that the JIMT-1mKate cells established tumors throughout the body. At the end of the study, the liver, lungs, kidney, adrenal glands, ovaries, eyes, and brain from each animal were harvested and then imaged in order to determine the presence and extent of disease. Representative fluorescent ex vivo images of excised organs from animals with tumors are shown in Figure 1c. Animals developed disease post-intracardiac inoculation in the lung, liver, ovary, the adrenal gland, brain, and kidney to varying degrees (Figure 1d). Tumors occasionally developed in areas such as the eyes and several lymph nodes, but less frequently. Imaging was optimized using a positive control ovary bearing JIMT-1mKate tumor tissue and a negative control ovary bearing JIMT-1 parental tumor tissue as shown in Figure 1e. Hematoxylin staining was performed on normal organs as well as tumor-positive organs. Representative images for an orthotopic tumor as well as the ovary and liver are shown in Figure 1f. Orthotopic tumors were typically poorly differentiated, with densely packed tumor cells and areas of necrosis. Tumors that developed in the adrenal gland and ovary after IC injection eventually took over the entire organ, pushing normal tissue to the periphery. In the lung, liver, brain, and kidney, multiple discrete tumors throughout the organ were observed. Regardless of organ type, the tumor within the organ had a higher cell density.

### 2.2. Assessing Architectural Features and Multiple Tumor Markers in JIMT-1 Tumors Grown Orthotopically and within Various Organs Following IC Injection

**Figure 2:**
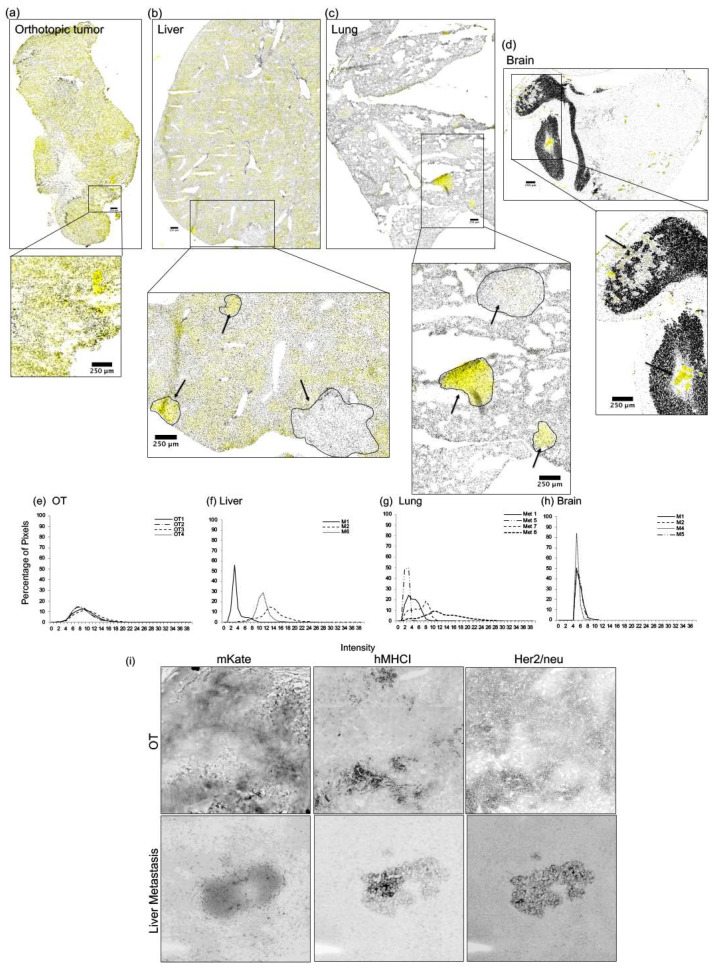
mKate, Her2/neu, and Hoechst nuclear dye were used to identify metastatic lesions in organ tissues subject to tumor mapping immunofluorescence. The location of metastatic lesions within normal tissues was identified using images of Hoechst 33342 nuclear dye (grey) and mKate (yellow). Although JIMT-1 cells were transduced with mKate, orthotopic tumors (**a**) and metastatic lesions (**b**–**d**) showed significant heterogeneity in mKate positivity when comparing lesions from different locations, as well as when comparing metastatic lesions within the same liver (**b**), lung (**c**), or brain (**d**) sections. mKate fluorescence was evaluated for heterogeneity by plotting the distribution of pixels across intensity levels from 0 to 255. When comparing entire tumor maps of orthotopic tumors or lesions of the brain, the distribution of pixels across intensity levels is similar for four animals (**e**,**h**). However, when comparing lesions within the liver and lungs, heterogeneity in mKate expression is observed (**f**,**g**). Bar = 250 µm. Orthotopic tumors and metastatic lesions from the liver were examined for the presence of mKate, hMHC1, and Her2/neu in order to determine how well each of these markers would be able to distinguish between human tissue and mouse tissue. The liver metastasis shown exemplifies heterogeneity in the staining of all three markers (**i**).

Serial sections of tumor-bearing organs collected from animals previously injected with JIMT-1mKate cells were prepared as described in Section 4. Solid tumors were easily identified within normal organ tissues after Hoechst staining, where the tumors typically appeared as clusters of larger and more disorganized cells. Initially, it was expected that mKate would be a suitable marker to identify tumor tissue within a normal organ. However, although solid tumors of the mammary fat pad (Figure 2a) and lesions in the brain (Figure 2d) had a homogenous expression of mKate throughout the tumor or across the lesions evaluated, surprisingly, mKate expression was heterogeneous between metastases within the liver (Figure 2b) and lung (Figure 2c), where some lesions were strongly positive for mKate expression and others were negative. It should be noted that JIMT-1mKate cells were clonally selected and characterized before use in these studies (see Section 4), and orthotopic tumors developing over a period of 30 days retain mKate expression suggesting that the variability of mKate expression observed in metastatic sites occurs as a result of clonal selection and/or growth phase of tumor cells. Furthermore, in the liver (Figure 2b), mKate fluorescence was observed dispersed throughout normal liver tissue, making it difficult, based on mKate alone, to discern normal versus tumor tissue. This was not due to auto-fluorescence in the liver as livers from control animals did not show fluorescence in the 588-range used to image mKate. Rather, the fluorescence in the 588-range seen in the livers of animals post-IC inoculation of JIMT-1mKate cells may have been due to destruction/metabolism of the JIMT-1mKate cells in the liver, an observation that has not been noted or reported previously.

Despite the heterogeneous expression of mKate, the average intensity of mKate fluorescence was evaluated and the results must be interpreted based on the presence of intact tumor cells or the presence of mKate from cells that were destroyed, and the marker was retained in the tissue. When comparing entire tumor maps of orthotopic tumors to tumors in the brain, the distribution of mKate intensity was similar (Figure 2e,h). However, within the liver and lungs, the unique distribution patterns for each tumor indicate heterogeneous expression of mKate from lesion to lesion.

**Figure 3:**
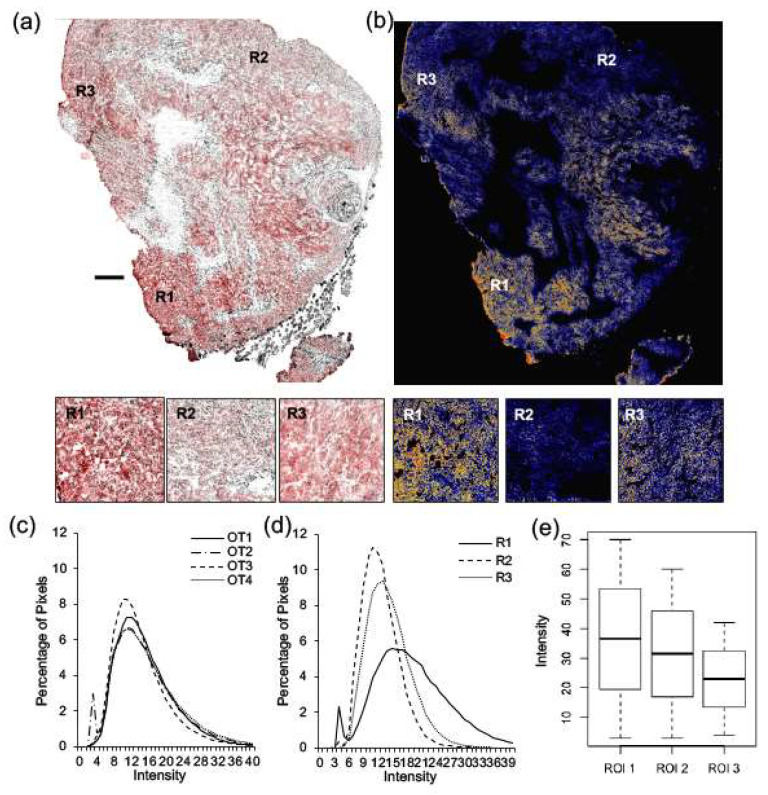
Significant intra-tumoral heterogeneity of Her2/neu expression is observed in orthotopic tumors. Tissue sections of orthotopic tumors were probed for Her2/neu expression and imaged for Her2/neu (red) and Hoechst (grey) (*n* = 4). A representative image of an orthotopic tumor is shown (**a**), selected regions from the tumor that represent high expression levels (R1), low expression levels (R2), and moderate expression levels (R3) are shown where grey represents nuclei, and red Her2/neu expression. A heat map was generated from the greyscale image of Her2/neu fluorescence (**b**, R1, R2, R3), where red represents strong staining, yellow moderate staining and blue weak staining. Her2/neu expression was evaluated for heterogeneity by plotting the distribution of pixels across intensity levels from 0 to 255 for four orthotopic tumors (**c**), and for three ROI within one orthotopic tumor (**d**). Boxplots were used to illustrate the distribution of pixels across intensity levels for three ROI within one orthotopic tumor, and a Kruskal–Wallis test provided a statistically significant *p*-value (<0.005). (**e**). Bar = 250 µm.

**Figure 4:**
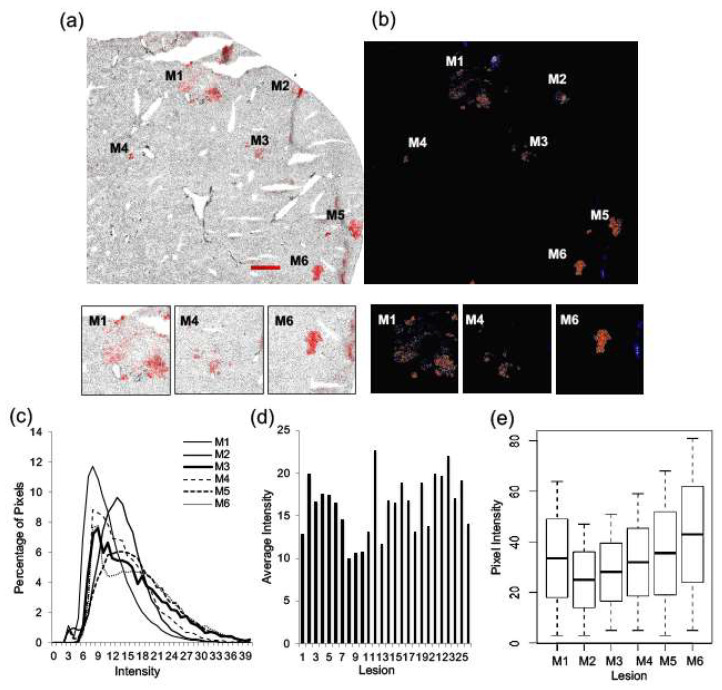
Significant inter-metastatic heterogeneity is seen for Her2/neu expression across liver lesions. Sections of liver tissue were probed for Her2/neu expression and imaged for Her2/neu (red) and Hoechst (grey) (*n* = 3). A representative image of a section of liver tissue is shown (**a**), and metastatic lesions are identified as M1 through to M6. Selected lesions that represent high expression levels (M6) and low expression levels (M1 and M4) are shown. A heat map was generated from the greyscale image of Her2/neu fluorescence (**b**). Her2/neu expression was examined by looking at the distribution of pixels across intensity levels from 0 to 255 for each lesion within the tissue (**c**). The average pixel intensity for each lesion within 3 liver samples from three animals was also plotted (**d**). Boxplots were used to illustrate the distribution of pixels across intensity levels for each lesion within one liver section, and a Kruskal–Wallis test provided a statistically significant *p*-value (<0.0001) (**e**). Bar = 250 µm.

**Figure 5:**
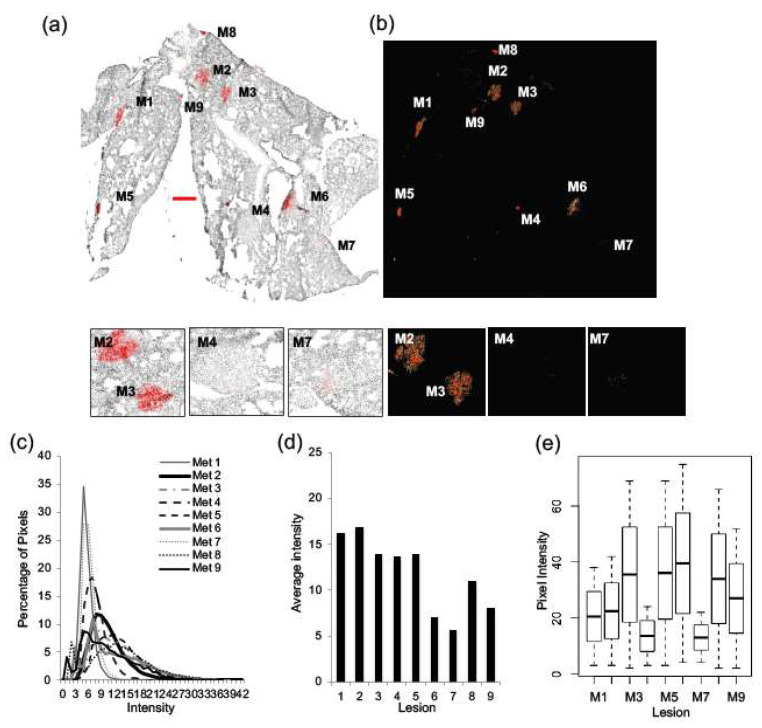
Significant inter-metastatic heterogeneity is seen for Her2/neu expression across lung lesions. Sections of lung tissue were probed for Her2/neu expression and imaged for Her2/neu (red) and Hoechst (grey) (*n* = 3). A representative image of a section of lung tissue is shown (**a**), and metastatic lesions are identified as M1 through to M9. Selected lesions from the lung that represent high expression levels (M2 and M3), as well as low expression levels (M4 and M7), are shown. A heat map was generated from the greyscale image of Her2/neu fluorescence (**b**). Her2/neu expression was examined by looking at the distribution of pixels across intensity levels from 0 to 255 in each of the lesions within the tissue (**c**). The average pixel intensity for each lesion was also calculated and plotted (**d**). Boxplots were used to illustrate the distribution of pixels across intensity levels for each lesion within one lung section, and a Kruskal–Wallis test provided a statistically significant *p*-value (<0.0001) (**e**). Bar = 250 µm.

**Figure 6:**
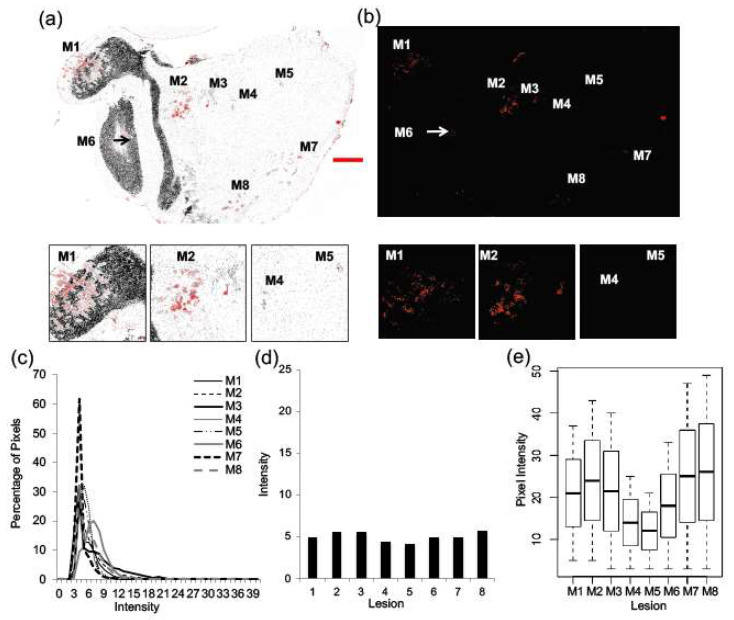
Inter-metastatic heterogeneity is not observed in Her2/neu expression across brain lesions. Tissue sections of the brain were probed for Her2/neu expression and imaged for Her2/neu (red) and Hoechst (grey) (*n* = 2). A representative image of a section of brain tissue is shown (**a**), and metastatic lesions are identified as M1 through to M8. Selected lesions from the brain are also shown (M1, M2, M4, and M5). A heat map was generated from the greyscale image of Her2/neu fluorescence (**b**). Her2/neu expression was examined by looking at the distribution of pixels across intensity levels from 0 to 255 for each lesion within the tissue (**c**). The average pixel intensity for each lesion was also plotted (**d**). Boxplots were used to illustrate the distribution of pixels across intensity levels for each lesion within one brain section, and a Kruskal–Wallis test provided a statistically significant *p*-value (<0.005) (**e**).

As an alternative marker for the selection of human tissue within mouse tissue, mammary fat pads and liver tissues were probed for human major histocompatibility marker 1 (hMHC1) (Figure 2i). Surprisingly, hMHC1 also showed considerable heterogeneity in expression across different tumors and could not be used as a selection marker. Finally, because JIMT-1 cells are Her2/neu-positive, Her2/neu expression was examined as a possible marker for tumor tissue identification within organs. Orthotopic tumors and livers from mice administered an IC injection of JIMT-1 cells were analyzed for Her2/neu expression, and as shown in Figure 2i, Her2/neu expression was also heterogeneous. This is further evaluated and confirmed in Figure 3, Figure 4, Figure 5 and Figure 6.

Due to the variability in expression of the three possible molecular markers, identification of metastatic lesions within organ sections was performed by careful examination of the section following use of a DNA stain (Hoechst 33342), as well as expression of mKate wherever possible.

### 2.3. Intra- and Inter-Metastatic Heterogeneity of Her2/Neu Expression

Tissue sections of orthotopic tumors (*n* = 4) as well as tumors arising in liver (*n* = 3), lung (*n* = 3), and brain (*n* = 2) were probed for Her2/neu. Representative images of an orthotopic tumor are shown in Figure 3. Selected regions from the tumor that represent high expression levels (R1), low expression levels (R2), and moderate expression levels (R3) of Her2/neu (red) overlayed on Hoechst images (grey) are also shown. A heat map was generated from the greyscale image of Her2/neu fluorescence to highlight the heterogeneity of Her2/neu expression levels (Figure 3b, R1, R2, R3). Her2/neu staining was evaluated for heterogeneity by plotting the distribution of pixel intensities for orthotopic tumor maps. The Her2/neu intensity distribution was similar for four OT tumors taken from four different animals (Figure 3c). However, when comparing regions of high to low expression within one OT tumor, intra-tumor heterogeneity is observed (Figure 3d). Boxplots illustrate the distribution of pixels (*p*-value < 0.005) (Figure 3e)

Sections of liver tissue with metastatic lesions were probed for Her2/neu (red) and stained with Hoechst (grey) (*n* = 3). A representative image of a section is shown in Figure 4a,b, and metastatic lesions are identified as M1 through to M6. Selected lesions that represent high expression levels (M6) and low expression levels (M1 and M4) are shown. Figure 4c illustrates the Her2/neu intensity levels for each lesion within the section evaluated. Each of the 6 lesions shown exhibit unique distributions, indicating heterogeneity in Her2/neu expression across different tumors within one liver. The average pixel intensity for each lesion within three liver samples from three animals was plotted and exemplifies this heterogeneity (Figure 4d). Boxplots depict the pixel intensity distributions for each lesion within one liver section (*p*-value < 0.0001) (Figure 4e). The data collectively illustrates significant inter-metastatic heterogeneity of Her2/neu expression from lesion to lesion in the liver.

Similarly, as shown in Figure 5, sections of lung tissue were probed for Her2/neu expression (*n* = 3). Significant inter-metastatic heterogeneity of Her2/neu expression was observed, where some lung lesions were strongly positive for Her2/neu and others were negative. Representative images of a section of lung tissue are shown (Figure 5a,b). Selected lesions from the lung that represent high expression levels (M2 and M3), as well as low expression levels (M4 and M7), are labeled. As observed in the liver, lung lesions exhibit unique intensity distributions (Figure 5c) and average pixel intensities (Figure 5d) that are quantitatively distinct with a significant *p*-value (<0.0001) (Figure 5e).

Brain tissues (*n* = 2) were similarly assessed and a representative image of a section of brain tissue is shown in Figure 6a, b, and metastatic lesions are identified as M1 through to M8. The distribution of Her2/neu staining intensity exhibits less variability than was observed in orthotopic, liver, and lung tumors. In contrast to the liver and the lung, brain metastases exhibited more homogenous but lower expression of Her2/neu, where all lesions were Her2/neu-positive, showing an average intensity level of 4.5 (Figure 6c–e).

### 2.4. Intra-Tumor and Inter-Metastatic Vascular Heterogeneity

**Figure 7:**
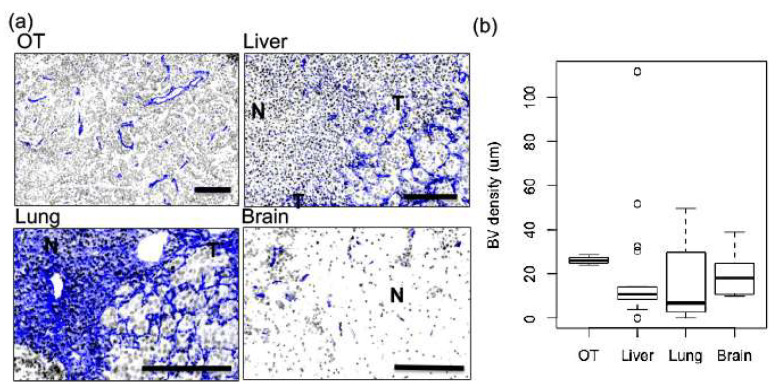
Vascular architecture and density show significant intra-tumoral and inter-metastatic heterogeneity. To examine vascular density, tissue sections were stained with Hoechst (grey) and CD31 (blue). The size and structures of blood vessels are shown in representative lesions from orthotopic tumors, lung, liver, and brain (**a**). Differences in vascular architecture can be appreciated between lesions (T), as well as between lesions (T) and their respective adjacent normal tissues (N). Bar = 100 µm. Vascular density was calculated using an automated algorithm that calculates the average distance of any given pixel in a region of interest to a CD31-positive pixel. The vascular density for each lesion was plotted as a box and whisker plot to illustrate the variability, and a Kruskal–Wallis test did not provide a statistically significant *p*-value (0.1983) (**b**).

To examine vascular characteristics within tumor tissue, tissue sections from orthotopic tumors, liver, lung, and brain were probed for CD31 (blue) and stained with Hoechst (grey). Figure 7a shows representative tumor tissues (T) in comparison to surrounding normal tissue (N) for the lung, liver, and brain. Considerable differences in vascular architecture can be appreciated between lesions located in different organs, as well as differences between tumor and surrounding normal tissues. Orthotopic tumors primarily have medium-sized vessels that are distinct from each other, while when present, vessels in lesions of the liver and the lungs appear as larger and more highly branched networks. The vessels of brain metastases are smaller compared to those in other organ metastases, but are enlarged when compared to surrounding normal brain tissue.

Vascular density was determined based on the average distance between a nuclei and its nearest CD31-stained object in an overlayed image and was plotted as a box and whisker plot to illustrate the variability of tumor blood vessel density in different organs as well as the range in densities found in metastases of the same organ (Figure 7b). Orthotopic tumors show the tightest distribution, while lesions of the liver, lung, and brain illustrate considerably more variability.

**Figure 8:**
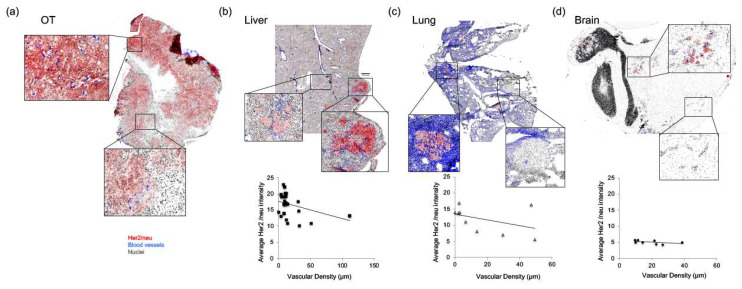
Tissue sections were simultaneously probed for Her2/neu expression (red) and CD31 (blue) from orthotopic tumors (**a**), liver (**b**), lung (**c**), and brain (**d**) tissues. Vascular density was quantified by calculating the average distance from each pixel to a CD31-positive object and correlated with average pixel intensity of the Her2/neu expression for liver (**b**), lung (**c**), and brain lesions (**d**).

Tissue sections were simultaneously probed for Her2/neu expression (red) and CD31 (blue) (Figure 8) to explore a possible correlative relationship between the two markers. Interestingly, in orthotopic tumors, vascular density and prominent vessels with large diameters are seen in regions of strong Her2/neu positivity, while few small vessels are seen in regions of low Her2/neu staining (Figure 8a). Vascular density was plotted against the average pixel intensity of the Her2/neu staining for liver (Figure 8b), lung (Figure 8c), and brain lesions (Figure 8d).

## 3. Discussion

Solid tumor models have been the foundation for preclinical drug efficacy studies and have certainly provided oncologists with agents that have benefited patients across cancers. However, subcutaneous and orthotopic models are limited in their translation to clinical outcomes. One explanation for this lack of translation from bench to bedside has to do with the heterogenous nature of metastatic disease. It has been well-established that the gene expression profile of a metastatic lesion can vary from the primary tumor [18,20,32,33,34,35]. If genetic heterogeneity is translated to a phenotypic heterogeneity, the result will influence drug or drug combination activity in the secondary tumor when compared to the primary tumor. Discordance in marker expression between primary and metastatic lesions has also been recognized clinically [36,53,54,55,56,57,58], but there remains a paucity of research into the extent and impact of this discordance on treatment. Furthermore, genotypic/phenotypic differences in tumors are in addition to other sources of heterogeneity arising from the variable microenvironment and immunological landscape of different organs hosting metastatic growth. Despite this knowledge and an awareness that effective treatments for disseminated, metastatic disease are most urgently required in the clinic, preclinical studies of drug efficacy are most often performed in homogenous tumors inoculated in single sites, typically as subcutaneous or orthotopic implants. Studies testing the efficacy of novel cancer therapeutics need to focus on targeting aggressive, heterogeneous, and metastatic disease. If this is accepted, then preclinical drug efficacy studies require models where metastatic spread has already occurred.

The current study focuses on a Her2/neu-positive cell line used to establish metastatic disease in immunocompromised animals. Organs were systematically excised and the expression of a number tumor and microenvironmental markers were evaluated for phenotypic heterogeneity from lesion to lesion. Significant heterogeneity in ECM proteins, hypoxia, vascular markers, as well as tumor markers was observed. Two well-studied markers (Her2/neu and CD31) are presented in the current report to show the substantial heterogeneity that exists in the tumor and the microenvironment when comparing metastatic lesions within the same organ and from organ to organ. These data support the conclusion that a well-designed preclinical study should evaluate drug candidates in multiple models, including models of established metastases, before moving on to clinical trials. If the drug candidate is effective in models of established metastases, that may translate to better clinical trial outcomes. If, however, the candidate drug fails preclinically using models of established metastasis, the investigator may choose to move on to a different candidate or examine the heterogenous variables that led to this failure so that drug delivery systems such as lipid nanoparticles may be designed to overcome these challenges. As an example, our interest is in utilizing a Her2/neu model as one to characterize the therapeutic benefit of Her2/neu-targeted combination therapeutics built using nanotechnology. The changes in Her2/neu expression and tumor microvasculature observed in the studies reported highlight how such drug combination products should be designed. Future studies should also be completed in immunocompetent animals to inform drug design that considers heterogeneity of immunological features of the disease.

We recognize that developing and using metastatic disease models is challenging for a number of reasons, including difficulty in monitoring disease progression over time, the fast growth patterns of xenograft models in mice compared with prolonged periods for tumor progression and growth in the clinic and the relatively limited number of models that metastasize from the original primary tumors as representations of true metastatic disease. Challenges of monitoring systemic disease can be overcome by using small animal imaging [59]. However, the use of these models is infrequent, primarily due to the need for specialized equipment, as well as the skills and expertise required to implement such experiments. We have demonstrated that it is relatively easy to implement an approach using tools that are more common to oncology research labs, such as a fluorescently tagged, commercially available cell lines, in vivo and ex vivo fluorescent imaging (FLI), multiplex immunohistochemistry (mIHC), and a semi-automated, open-access digital analysis tool (image j) [60].

While it has been shown both preclinically and clinically that there can be changes in Her2/neu expression in metastases compared with treated and untreated primary tumors [36,48,55,56,58], we were surprised at the extent of variability in Her2/neu expression when comparing metastases even within the same organ. This finding is particularly important as the anti-Her2/neu therapeutic antibody Herceptin is the gold standard of treatment for women expressing this marker in primary tumor biopsies, but the disease can recur metastatically in these patients. Patients treated with Herceptin are living longer as Herceptin eradicates primary tumors, however the number of women with brain metastases has increased [61,62,63,64]. If, as our results indicate, metastases are significantly variable in Her2/neu expression, this explains why Her2/neu-positive patients treated with Herceptin have recurring metastatic disease. Our results suggest that research efforts should be directed towards designing a combination of Her-2/neu-targeting with other agents that can treat tumors that lack Her-2/neu expression. These combinations should be tested preclinically in models of established metastases. This is also likely to be important for other tumor markers where targeted treatment is available clinically.

We showed that there is heterogeneity in CD31 expression, indicating heterogeneity in blood vessel structure when comparing lesions in different organs as well as lesions within the same organ. This finding is also supported by studies that have demonstrated variability in vascular characteristics between primary tumors and metastatic lesions [65]. For example, Jubb et al. show that the proportion of mature vessels was higher in brain metastases than in matched, non-small-cell lung cancer primary tumors [66]. Variability in vessel density, architecture, or maturity from lesion to lesion can have a significant impact on drug delivery and distribution. Previously, our lab demonstrated that the drug docetaxel has variable drug efficacy depending on the location of disease burden (orthotopic, ascites, and systemically disseminated metastases), and these differences may be overcome by using cytotoxic therapy in combination with a targeted agent [67]. Furthermore, research shows that there can be variable amounts of drug in different metastases even within the same organ [29,30,46,47,48,68,69]. At this stage, it is unclear how the relationship between vascularity and drug distribution will vary from tumor to tumor or between metastases, but it is clear that this will impact therapeutic outcomes, and can be evaluated preclinically using models of established metastases such as the one described.

Interestingly, there seems to be a relationship between Her2/neu expression and vascular density (Figure 8). We were unable to explore the relationship fully in these studies, but the consequences are significant. Novel metrics able to identify and compare hotspots of expression or architectural features will be best suited to characterize these types of correlations. Spatial autocorrelation is one example of a metric that may be valuable in this type of work, such as GetisOrd Gi hotspot analysis used in landscape ecology [70,71,72,73]. Another possibility is to develop deep learning algorithms to analyze multiplex imaging of tumor maps [74,75,76,77]. Regardless, differences in vessel architecture may have significant impacts on blood flow, hypoxia, and ultimately drug distribution and efficacy [45]. It is known that monoclonal antibodies such as Herceptin have trouble accessing lesions due to vascular variability [78,79,80]. If there is a correlation between low Her2/neu expression and poor vascularity, as shown in this report, the efficacy of Herceptin would be attenuated, leading to recurrence and metastatic spread. In this case, use of small molecule inhibitors in combination with monoclonal antibodies and/or drug delivery systems could help improve access and efficacy and should be evaluated preclinically using models of established metastases.

These studies describe profound variability of tumor and microenvironment features between lesions in different organs and between lesions localized within the same organ in a preclinical model of established metastasis. Heterogeneity was significant for a clonally selected expression marker used to track and trace neoplastic cells (mKate), the expression of a therapeutic target (Her2/neu), as well as the bedrock architecture of the microenvironment itself—the vascular density and structure (CD31). This data demonstrates the heterogenous nature of aggressive metastatic disease phenotypes and supports the critical need to investigate candidate drugs in multiple disease models that recapitulate this heterogeneity. Intracardiac inoculations of a defined cell line may serve as one such model to examine drug efficacy in systemic disease, with the added ability to screen for and identify possible detrimental features of drug candidates such as low distribution. Using metastatic models such as the one described will not only improve the ability of preclinical studies to predict clinical success of experimental drugs, it will also help to concentrate research efforts on those drugs with the ability to engender better outcomes for patients with disseminated disease.

## 4. Materials and Methods

### 4.1. Cell Lines and Culture

JIMT-1 cells were a gift from Dr. Jorma Isola (Tampere University and Tampere University Hospital, Tampere, Finland). Cells were resuspended in freezing media (10% DMS0 in FBS) and slowly frozen in Nalgene^®^ 1 °C freezing containers (Rochester NY, USA) containing 100% isopropanol at −80 °C for 24 h, before storage in liquid nitrogen. Frozen cells were quickly thawed at 37 °C, centrifuged to remove freezing media, plated, and passaged twice before use in experiments. Cells were maintained in DMEM/high glucose supplemented with L-glutamine (2 mMol/L; DMEM and L-glutamine from Stem Cell Technologies, Vancouver, British Columbia, Canada), 5 mM penicillin/streptomycin (Stem Cell), and 10% fetal bovine serum (FBS) (Hyclone, Logan, UT, USA). All cells were maintained at 37 °C and 5% CO_2_ in a humidified atmosphere and allowed to undergo no more than 20 passages. Cells were maintained in the absence of penicillin and streptomycin and screened for mycoplasma prior to preparing the stock of cells that were frozen for future use in animal experiments.

### 4.2. Lentivirus Transfections

JIMT-1mKate cells were generated by lentiviral transfection of JIMT-1 cells with the gene encoding mKate2 [81]. The pFUKW transfer plasmid had been generated by inserting the mKate gene, obtained from the pmKate2-N plasmid (Evrogen, Saphire North America, Ann Arbor, MI, USA), into a pFUW vector backbone [82]. Lentivirus particles were generated by combining pFUKW with the envelope plasmid pVSV-G (Clontech, Mountain View, CA, USA) and the packaging plasmid pDeltaR8.91 [83] in HEK 293T cells. Viral supernatant was harvested, filtered, and subsequently used to infect target JIMT-1 cells. Resulting JIMT-1mKate cells were sorted by FACS for red fluorescence and the top 12% were used for subsequent experiments.

### 4.3. Animal Studies

All animal studies were conducted in accordance with institutional (University of British Columbia guidelines for humane animal treatment and according to the current guidelines of the Canadian Council of Animal Care (protocol number A14-0290). Mice were maintained at 22 °C in a 12 h light and dark cycle with ad libitum access to water and food. The studies used female NCr nude mice weighing between 18 and 25 g, which were obtained from Taconic (Oxnard, CA, USA) and maintained in an SPF-Facility. Animals were housed in groups of 4 or 5. Long-term survival was determined based on the time in days when mice were terminated due to tumor ulceration, the presence of tumors exhibiting volumes in excess of 500 mg, and/or signs of deteriorating animal health requiring euthanasia, as defined by a health monitoring standard operating procedure. Animals that did not develop tumors were excluded from the study.

To initiate orthotopic disease, 2 × 106 JIMT-1mkate cells were inoculated into the mammary fat pad in a volume of 50 µL media using a 28-gauge needle. For the intracardiac inoculation of cells, animals were anesthetized using isofluorane and positioned so that a 26-gauge needle attached to a 1 mL tuberculin syringe could be inserted at a 30-degree angle immediately caudal to the xyphoid process. The needle was aimed towards the left shoulder. Slight negative pressure was placed on the syringe upon entry and the needle was slowly moved forward until blood appeared in the hub of the needle. Once the needle was in the correct position, 1.8 × 105 JIMT-1mkate cells in a volume of 100 µL of media were slowly (over 30 to 60 s) injected. Animals were monitored for tumor growth, body weight, and health status. Tumor growth for both OT and IC models was monitored using the Maestro imaging system (Perkin Elmer, MA, USA), as described below. Animal body weights were measured every Monday and Friday.

### 4.4. Maestro Imaging

Imaging was performed once per week to monitor tumor growth and localize sites of metastasis. Fluorescent images of the whole mouse body were obtained using the Maestro in vivo fluorescence imaging system (Perkin Elmer, MA, USA). Mice were anaesthetized using isoflurane, and photographic and fluorescent images were captured of the location of mKate under the following conditions: “Blue” filter model, excitation filter range 455 nm (435–480 nm), emission filter range 490 nm long-pass, acquisition setting, 500–720 nm in 10 nm steps. Both the auto-fluorescence of mice and mKate fluorescence were obtained and then unmixed using Maestro spectral software. Ex vivo imaging of mKate for the liver, lungs, kidney, adrenal glands, ovaries, and brain was performed for each animal.

### 4.5. Tissue Collection and Immunofluorescence

Animals were sacrificed by CO_2_ asphyxiation, tumors and organs were excised, cut in half and embedded in OCT, and immediately placed on dry ice. Samples were stored at −80 °C. Ten, 10 μm tumor cryosections were cut using a Cyrostar HM560 (Microm International, Waldorf, Germany), air-dried, and imaged for exogenous marker native fluorescence, mKate (visualized at 633 nm). Sections were fixed in 50% (*v*/*v*) acetone/methanol for 10 min at room temperature and subsequently stained for: (1) CD31 using a rat monoclonal anti-mouse CD31 antibody (BD Pharmingen) and Alexa 647 secondary antibody (Invitrogen), (2) Her2/neu using Herceptin, a monoclonal anti-human Her2/neu antibody (Genentech/Roche), followed by an Alexa 546 secondary antibody (Invitrogen), and (3) cell nuclei using Hoechst 33342, Bis-Benzimide (Sigma) (8 μg/mL at 37 °C), for 30 min.

### 4.6. Image Acquisition and Analysis

The imaging system consists of a robotic fluorescence microscope (Zeiss Imager Z1), a cooled, monochrome CCD camera (Retiga 4000R, QImaging, Surrey, BC, Canada), a motorized slide loader and x-y stage (Ludl Electronic Products, Ltd., NY, USA), and customized NIH-ImageJ software, described in detail elsewhere [84,85]. The system allows adjacent microscope fields of view to be imaged and automatically tiled to produce a montage of the entire tumor cryosections at a resolution of 0.75 μm/pixel for qualitative and quantitative analysis. All variables stained on the same section were imaged separately using the monochrome camera and subsequently overlaid and aligned for analysis and to generate false-color images using ImageJ software applications (NIH ImageJ). Images were screened manually to identify regions of interest (ROI) (i.e., tumor tissue versus normal tissue) and artefacts of processing. ROI were outlined and cropped, and artefacts were removed before further analysis. Intensity histograms were generated for a selected ROI on a tumor or organ map by plotting the distribution of pixels across intensity levels from 0 to 255 as a percent of total pixels in the ROI. Cellularity was determined by thresholding the Hoechst layer and masking nuclei using the built-in particle counting plugin. From the masked layer, the area covered by Hoechst-positive pixels was determined and divided by the total area of the ROI in order to obtain the percent area covered by nuclei, and the average intensity of pixels in the ROI was calculated. Blood vessel (BV) density was determined as the average distance of pixels within the ROI to their nearest CD31-positive object.

### 4.7. Statistical Analyses

Boxplots were used to illustrate the distribution of pixel intensities across lesions. A Kruskal–Wallis nonparametric ANOVA test was used to compare the distributions. Differences were considered to be statistically significant at *p* < 0.05. All analyses were performed using R Studio (version 1.0.136).
